# Supplementary material for: Predicting prostate cancer metastasis in Ghana: Comparison of multiparametric and PSA models
Source: PLoS One. 2025 May 28;20(5):e0323180. doi: 10.1371/journal.pone.0323180 (PMC12119020; doi:10.1371/journal.pone.0323180)
Supplement: S1 Fig — (DOCX) [file pone.0323180.s001.docx]

**Fig 1 Receiver –Operator –Characteristic Curves; and Corresponding Sensitivity/Specificity Curves for the Models for Detecting Metastasis in Prostate Cancer(The Multiparametric model). The Youden Index point is indicated as well (0.40)**

logit(**MET_CD**) = −13.5227+0.876⋅ACT+3.947⋅DRE_CD+0.00238⋅PSA+0.389⋅ISUP – (1)

Where:

- logit(Mets) represents the natural logarithm of the odds of the outcome variable pp. in this case, the risk of metastasis.
- The coefficients are rounded to three decimal places for simplicity.
- The intercept term _cons is −13.5227−13.5227 based on the actual value.
- The coefficients for the variables are 0.8760.876 for ACT, 3.9473.947 for DRE_CD, 0.002380.00238 for PSA, and 0.3890.389 for ISUP, based on the actual values of the odds ratios.

This equation describes how changes in the predictors (ACT, DRE_CD, PSA, ISUP) influence the log-odds of the outcome, with all other variables held constant.

The full multiparametric model equation is below.

**log(odds of MET_CD) = -12.809 + (0.003 * AGE) - (0.067 * MAR_CD) + (0.073 * ETH_CD) + (0.875 * ACT) - (0.945 * BMI_CD) - (0.314 * FMH) + (0.399 * ALC) - (1.142 * TBC) + (0.203 * LOC_CD) + (3.946 * DRE_CD) + (0.002 * PSA) + (0.386 * ISUP) – (Full).**
