# Supplementary material for: Predicting prostate cancer metastasis in Ghana: Comparison of multiparametric and PSA models
Source: PLoS One. 2025 May 28;20(5):e0323180. doi: 10.1371/journal.pone.0323180 (PMC12119020; doi:10.1371/journal.pone.0323180)
Supplement: S2 Fig — (DOCX) [file pone.0323180.s002.docx]

**Fig 2 Receiver –Operator –Characteristic Curves; and Corresponding Sensitivity/Specificity Curves for the Models for Detecting Metastasis in Prostate Cancer (PSA alone model). The Youden Index point is 0.25**
