# Supplementary material for: Predicting prostate cancer metastasis in Ghana: Comparison of multiparametric and PSA models
Source: PLoS One. 2025 May 28;20(5):e0323180. doi: 10.1371/journal.pone.0323180 (PMC12119020; doi:10.1371/journal.pone.0323180)
Supplement: S3 Fig — (DOCX) [file pone.0323180.s003.docx]

**Fig 3: The combined ROC curves for the Multiparametric Model and the PSA-Alone Model are now displayed on the same axes. The Multiparametric Model demonstrates superior performance with a higher AUC (0.9718) compared to the PSA-Alone Model (0.7379), showcasing its better discriminatory power in predicting prostate cancer metastasis.**
