# Supplementary material for: Predicting prostate cancer metastasis in Ghana: Comparison of multiparametric and PSA models
Source: PLoS One. 2025 May 28;20(5):e0323180. doi: 10.1371/journal.pone.0323180 (PMC12119020; doi:10.1371/journal.pone.0323180)
Supplement: S4 Fig — (DOCX) [file pone.0323180.s004.docx]

**Fig 4: The bar chart visualizes the sensitivity, specificity, and AUC for each subgroup from the analysis. Each metric is represented by a different bar, allowing for a side-by-side comparison across subgroups.**
