# Supplementary material for: Predicting prostate cancer metastasis in Ghana: Comparison of multiparametric and PSA models
Source: PLoS One. 2025 May 28;20(5):e0323180. doi: 10.1371/journal.pone.0323180 (PMC12119020; doi:10.1371/journal.pone.0323180)
Supplement: S5 File — (DOCX) [file pone.0323180.s005.docx]

**LEGEND/LIST OF ABBREVIATIONS AND THEIR MEANINGS**

1. **MET_CD**: This represents the binary outcome variable, likely indicating the presence or absence of metastasis of the prostate cancer.
2. **AGE**: Age of the individual at the time of assessment.
3. **AGE_CD**: Categorized or coded version of age.
4. **MAR_CD**: Marital status code, potentially representing different categories of marital status (e.g., married, single, divorced).
5. **ETH_CD**: Ethnicity code, indicating the individual's ethnic or racial background.
6. **SES**: Socioeconomic status, representing the individual's social and economic standing within society.
7. **ACT**: Activity level or physical activity status.
8. **BMI_CD**: Body Mass Index (BMI) code, representing different categories of BMI (e.g., underweight, normal weight, overweight, obese).
9. **FMH**: Family medical history, indicating whether the individual has a family history of a particular condition or disease.
10. **ALC**: Alcohol consumption status or alcohol intake level.
11. **TBC**: CIGARRETTE SMOKING, PRESENT OR PAST
12. **LOC_CD**: Location code, potentially indicating the geographic or residential location of the individual.
13. **DRE_CD**: Digital Rectal Examination (DRE) code, representing the results or findings of a digital rectal exam.
14. **PSA**: Prostate-specific antigen (PSA) level, a biomarker used in prostate cancer screening and diagnosis.
15. **ISUP**: International Society of Urological Pathology (ISUP) grade, indicating the grade of prostate cancer based on histopathological examination.

## **3.13 The Variable Coding Process**

**Variables and Coding Plan: A. Independent Variables:**

1. **Age and Age-Categories:** Age was coded numerically. Age-categories were created, such as <45 (1), 45 to 54 (2), 55 to 64 (3), .65 to 74 (4), 75 to 84 (5). 85+ (6).
2. **Ethnicity:** Four major groups coded as Akan (1), Ga (2), Ewe (3), and Northern (4). This was limited to 4 only, for Ghanaians due to the quality of data we had. Additional codes for Other West African (5), Other Non-West African African (6), and European/Asian/American (7).
3. **Occupation and Marital Status:** Coded as Sedentary (1), Manual (2), Sporting (3), and Marital status as Single (0) or Married (1).
4. **BMI and BMI Categories:** BMI calculated and coded. Categories: Underweight (1), Normal (2), Overweight (3), Obese/Morbidly Obese (4-6).
5. **Alcohol Use, Tobacco Use, Family History, and Co-morbidities:** Coded as Yes (1) or No (0).
6. **Geographical Location and Nationality:** Location coded as Urban (1), Periurban (2), Rural (3). Nationality encompassing different groups.

**B. Dependent Variables:**

1. **DRE Stage at Diagnosis:**

**T1 to T2C = Localised Disease (coded as 1)**

**T3A, T3B , T4 WITH NO METASTASIS =Locally Advanced Disease (coded as 2)**

**ANY T STAGE WITH METASTASIS ANYWHERE = Advanced/Metastatic Disease. (coded as 3)**

1. **DRE T-STAGE RISK Categories:** Stages coded and categorized: T1-T2A =Low risk (1); T2B AND T2C; = Intermediate risk (2),

T3 and T4 = high risk (3)

1. **PSA at Diagnosis:** Coded as continuous numerical variable.
2. **PSA at Diagnosis, categories:** **Low risk:** PSA < 4 ng/Ml; given that it is localised disease (1), **Intermediate risk:** PSA 4 - 10 ng/Ml; given that it is localised disease (2), **High risk:** PSA > 10 ng/Ml; given that it is localised disease (3)
3. **Gleason Sum Score and ISUP Grade:** Gleason Score was converted to ISUP grades 1, 2, 3, 4, and 5; and coded categorically, as follows: -

ISUP 1; given that it is localised disease = Low-Risk (1):

ISUP 2 and 3; given that it is localised disease = Intermediate-Risk (2),

ISUP 4 and 5; given that it is localised disease = High-Risk (3).

1. **OVERALL RISK CATEGORY:**

**Overall**  **Low-Risk:** for those with only low risk for all three parameters; PSA Low + DRE Stage 1-2 + ISUP Grade 1 (1)

**Overal Intermediate-Risk:** all three parameters, PSA, DRE, ISUP Intermediate risk; or two intermediate risk in the presence of one low risk, or one intermediate risk in the presence of two low risk (2)

**High-Risk:** once any one of the parameters PSA, DRE or ISUP puts the patient at high risk level, it is an overall high risk category; coded as (3) in the analysis.

1. **Metastasis:** Coded as binary: Yes (1) or No (0).
