# Supplementary material for: Predicting prostate cancer metastasis in Ghana: Comparison of multiparametric and PSA models
Source: PLoS One. 2025 May 28;20(5):e0323180. doi: 10.1371/journal.pone.0323180 (PMC12119020; doi:10.1371/journal.pone.0323180)
Supplement: Attachment — Submitted filename: RESPONSE TO REVIEWERS CONCRNING DATA AVAILABILTY.docx [file pone.0323180.s007.docx]

Frank Obeng

Department of Surgery, Urology Unit
University of Health and Allied Sciences, Ho
Email: [fobeng@uhas.edu.gh](mailto:fobeng@uhas.edu.gh)
Telephone: +233 24 441 9607
13/02/2024

Paula Katrina A. Maderazo
PLOS ONE Editorial Office

Subject: Response to Data Availability Request for Manuscript PONE-D-24-36914R1

Dear Ms. Maderazo,

Thank you for your email regarding our manuscript, "Predicting Prostate Cancer Metastasis in Ghana: Comparison of Multiparametric and PSA Models" (PONE-D-24-36914R1). We appreciate the opportunity to address the data availability concerns.

We would like to clarify that the data from this study are subject to ethical and legal restrictions imposed by SGMC Ghana, which owns the original dataset. Due to institutional policies on patient confidentiality and data protection, SGMC Ghana has determined that the dataset cannot be publicly shared. This restriction is in place to protect potentially sensitive patient information and is in compliance with local ethical guidelines.

Requests for access to the data may be submitted for consideration by SGMC Ghana. Interested researchers should direct their inquiries to the following contact:

**SGMC Ghana Data Access Committee**
**Email:** *Contact* Details · East Legon Hills, Greater Accra · P. O. Box MD 1879 Madina Accra · info@*sgmcltd*.com · +233 262 253 328 · +233 506 735 186 · +233 307 032 133.

THE AUTHORS HAVE A PERMISSION FROM THE SGMC TO SHARE THE DATA WITH THE JOURNAL UPON REASONABLE REQUEST, ONLY AFTER THE MANUSCRIPT HAS BEEN PUBLISHED.

We have included this information in the 'Enter Comments' section of the online submission form as requested. Please let us know if further clarification is needed. We appreciate your guidance and look forward to proceeding with the review process.

Thank you for your time and consideration.

Best regards,

Dr. Frank Obeng
Department of Surgery, Urology Unit
University of Health and Allied Sciences, Ho
Email: [fobeng@uhas.edu.gh](mailto:fobeng@uhas.edu.gh)
Telephone: +233 24 441 9607

The de-identified dataset from this study is subject to ethical and legal restrictions imposed by SGMC Ghana, which owns the original data and has chosen to keep it confidential. As a result, access to the dataset is restricted to authorized personnel and cannot be publicly shared. Any requests for data access must be directed to SGMC Ghana for review and approval.

THE AUTHORS HAVE A PERMISSION FROM THE SGMC TO SHARE THE DATA WITH THE JOURNAL UPON REASONABLE REQUEST, ONLY AFTER THE MANUSCRIPT HAS BEEN PUBLISHED.

For inquiries regarding data access, please contact:

**SGMC Ghana**
**Email:** *Contact* Details · East Legon Hills, Greater Accra · P. O. Box MD 1879 Madina Accra · info@*sgmcltd*.com · +233 262 253 328 · +233 506 735 186 · +233 307 032 133
